# Growth Media Affect Assessment of Antimicrobial Activity of Plant-Derived Polyphenols

**DOI:** 10.1155/2018/8308640

**Published:** 2018-05-09

**Authors:** Xin Xu, Zhen M. Ou, Christine D. Wu

**Affiliations:** ^1^Department of Pediatric Dentistry, College of Dentistry, University of Illinois at Chicago, Chicago, IL, USA; ^2^State Key Laboratory of Oral Diseases & National Clinical Research Center for Oral Diseases, West China Hospital of Stomatology, Sichuan University, Chengdu, China

## Abstract

This study aimed to investigate the effects of different microbial growth media on the laboratory assessment of antimicrobial activity of natural polyphenolic compounds. The inhibition of the tea polyphenol EGCG on growth of selected oral microorganisms was evaluated in complex media and a protein-free chemically defined medium (CDM). Other antimicrobial agents (polyphenolic grape seed extract, plant alkaloid berberine, methyl salicylate, and chlorhexidine gluconate) were also tested in the study. The presence of proteins and their effects on the antimicrobial activity of EGCG were investigated by the addition of BSA to the CDM. The MICs of EGCG against test oral microorganisms were 4 to 64 times higher in complex media than in CDM. The polyphenolic grape seed extract exhibited similar discrepancies. However, the MICs of the nonpolyphenolic compounds (berberine, methyl salicylate, and chlorhexidine) were not significantly different between the two growth media. The MIC of EGCG against* S. mutans* UA159 in CDM with added BSA was 16 times higher than that in CDM alone. Therefore, nonproteinaceous CDM should be used to avoid interference of proteins with the active ingredients when testing the antimicrobial activity of plant-derived polyphenolic compounds against microorganisms. This will also minimize the discrepancies noted in results obtained by different investigators.

## 1. Introduction

Plant-derived polyphenolic compounds have been reported to have a wide range of biological activities, among which are their antimicrobial and antibiofilm activities against a wide range of microorganisms [1–5]. Over the years, our laboratory, along with many others, has evaluated the inhibitory effects of these compounds against many oral pathogens [6–9]. Tea (infusion of dried leaves of* Camellia sinensis*) is the most popular and widely consumed beverage in the world today [10]. Its polyphenolic component has been reported to possess antioxidant, antimicrobial, antimutagenic, antidiabetic, hypocholesterolemic, anti-inflammatory, and cancer-preventive properties [11–14]. Many researchers have demonstrated that tea and its polyphenols inhibited growth, acid production, metabolism, and glucosyltransferase (GTF) activity of cariogenic mutans streptococci and other dental plaque bacteria [15–18]. Its anticariogenicity has also been demonstrated in humans and experimental animals [15, 19–21]. Our previous studies have shown that epigallocatechin gallate (EGCG), the antimicrobial monomeric component of tea catechins (the major polyphenolic component in tea), exhibited a wide range of effects on* Streptococcus mutans*, particularly on virulence factors associated with its acidogenicity and acidurity [22]. EGCG also suppressed* gtf* gene expression in* S. mutans* and decreased the synthesis of extracellular polysaccharides (EPS), thus inhibiting cell adherence and biofilm formation [23].

In an attempt to compare antimicrobial data of tea polyphenols obtained among different investigators, we noticed that significant differences existed in reported antimicrobial activity, for example, MIC values, depending on the types of growth media used for their antimicrobial evaluations [22]. For testing the antimicrobial activity of polyphenols, ready-to-use complex media such as brain heart infusion (BHI) or tryptone broth are commonly used. These media contain high amounts of animal proteins and nutrients. Because polyphenols including EGCG have been reported to bind proteins [24], we hypothesized that protein components in complex media may affect the results of antimicrobial activity assessment of polyphenolic compounds.

In this study, the antimicrobial activity of EGCG on the growth and viability of representative oral microorganisms associated with dental caries, periodontal, endodontic, and oral fungal infections was evaluated in both complex and chemically defined protein-free media. The effects of MICs on the growth and viability of test organisms were compared. In addition, other natural antimicrobial compounds, including the polyphenolic grape seed extract, berberine (a plant alkaloid isolated from* Hydrastis canadensis*), and methyl salicylate (a plant essential oil also known as wintergreen oil), were also examined. Chlorhexidine gluconate, a cationic antimicrobial chemical used in mouth rinses, was used as a positive control.

## 2. Materials and Methods

Test bacteria included:* S. mutans* UA159,* S. mutans* ATCC 25175,* S. mutans Ingbritt*,* Streptococcus gordonii* ATCC 49818,* Streptococcus sanguinis* ATCC 10556,* Streptococcus sobrinus* ATCC 6715),* Enterococcus faecalis* ATCC 29212, and* Aggregatibacter actinomycetemcomitans* ATCC 43718. Both BHI media (Difco Laboratories, Detroit, MI, USA) and a modified protein-free chemically defined medium (CDM) were used [25]. Test* Candida *species included* Candida albicans* SC5314,* C. albicans* ATCC 10231, and the non-albicans* Candida* species* Candida glabrata* ATCC 66032,* Candida tropicalis* ATCC 13803,* Candida parapsilosis* ATCC 22019,* Candida kefyr* ATCC 46764, and* Candida krusei* ATCC 14243. These were grown in Sabouraud Dextrose Broth (SDB, Difco Laboratories) and RPMI 1640 medium (Difco Laboratories). SDB is a complex medium equivalent of BHI, and RPMI 1640 is a chemically defined medium containing no proteins, lipids, or growth factors. All test organisms were incubated in an anaerobic chamber (37°C, 10% H_2_, 5% CO_2_, and 85% N_2_; Forma Scientific, Inc., Marietta, OH, USA). Grape seed extract (GSE, 97.8% Proanthocyanidins; Polyphenolics, Inc., Madera, CA), epigallocatechin gallate from green tea (EGCG, 95% HPLC; Sigma-Aldrich Corp., St. Louis, MO, USA), berberine chloride (98% TLC, Sigma-Aldrich Corp.), methyl salicylate (≥99% GC, Sigma-Aldrich Corp.), and chlorhexidine gluconate (Sigma-Aldrich Corp.) were prepared in sterile deionized water at different test concentrations by a microdilution method [22, 26].

The MICs of test agents against the microorganisms were determined in 96-well microtiter plates as described previously [22, 26]. Each well contained test microorganisms (1.0 × 10^5^ CFU/mL of streptococci; 1.0 × 10^6^ CFU/mL of* E. faecalis* or* A. actinomycetemcomitans*; or 2.5 × 10^3^ CFU/mL of* Candida* species) and the respective growth medium containing the twofold-diluted test agent. Controls included inoculated growth medium without the test agent, uninoculated growth medium with the test agent, and inoculated medium with chlorhexidine gluconate. All plates were incubated anaerobically for 48 h, and growth was determined spectrophotometrically at 550 nm (PowerWave 200, Bio-Tek Instruments, Winooski, VT, USA). The MIC_90_ was defined as the lowest concentration of test agent demonstrating more than 90% of growth inhibition compared with the growth control. The minimum bactericidal/fungicidal concentrations (MBCs/MFCs) were defined as the lowest concentrations of test agents needed to kill 99.9% of bacterial/fungi [22, 27].

The effect of protein on the antimicrobial activity of polyphenolic compounds was also investigated by the addition of bovine serum albumin (BSA) to the assay mixtures. All MIC data were reported as the median of at least three independent tests. The fold change in MIC (complex medium versus CDM) of each test compound was calculated. The median fold change in MIC was used for the comparison of the effects of growth media on the antimicrobial efficacy of test compounds against selected oral microorganisms [28].

All experiments were performed at least 3 separate times. Statistical analysis of data was performed with SPSS® (version 18.0 for Windows; SPSS Inc., Chicago, IL, USA) using Student's paired *t*-tests to compare difference between CDM group and BHI group of each test agent. Data were considered significantly different if the 2-tailed *p* value was <0.05.

## 3. Results

**Figure 1:**
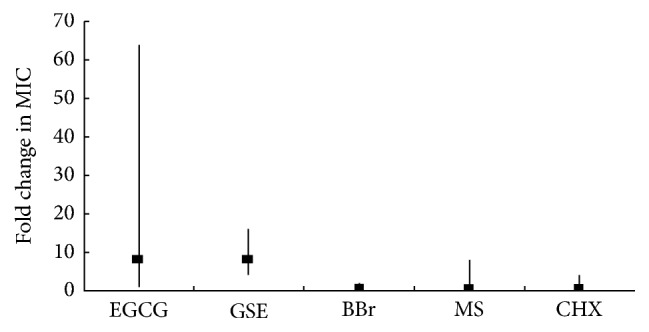
The effects of different growth media on the antimicrobial efficacy of test compounds against selected oral microorganisms. The vertical lines represent the range of the fold change in MIC (complex medium versus CDM). Square dots represent the median fold change in MIC. The antimicrobial efficacy of EGCG and grape seed extract (GSE) was significantly reduced in complex medium, while the efficacy of berberine chloride (BBr), methyl salicylate (MS), and chlorhexidine gluconate (CHX) was not significantly influenced by the growth medium.

The MICs of EGCG against the test streptococci grown in CDM ranged from 9.8 *μ*g/mL to 78 *μ*g/mL, and those against* A. actinomycetemcomitans* and* E. faecalis* were at 39 *μ*g/mL (Table 1). However, the MIC values of EGCG when assessed in BHI medium were 4 to 64 times higher than those determined in CDM (Table 1, Figure 1). The MICs for* Candida* species were much lower in the defined medium, RPMI 1640, than the values observed in the complex medium, SDB (Table 2). EGCG was bactericidal against all test bacteria, and the MBC values in BHI were similarly higher than those in CDM (Table 3). Of note, no significant discrepancies in MFC values of EGCG against Candia species were observed between RPMI 1640 and SDB, because EGCG was not fungicidal against most of the* Candida* species at the highest concentrations tested in this study (1250 *μ*g/mL) (Table 4).

**Figure 2:**
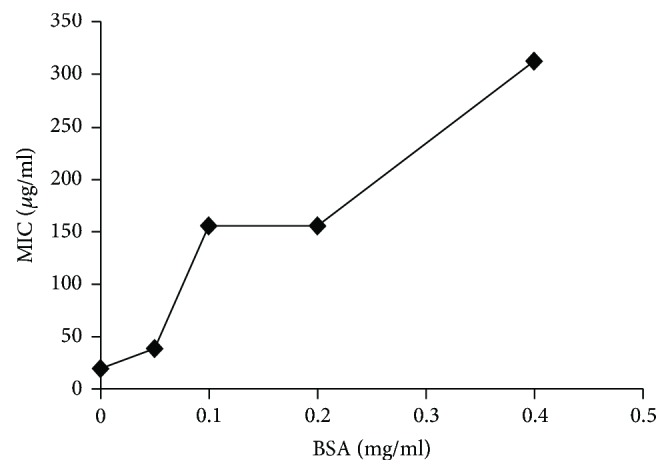
Effect of bovine serum albumin on the antimicrobial activity of EGCG against* S. mutans* UA159. A dose-dependent increase in the minimum inhibitory concentration (MIC) of EGCG was observed with the addition of BSA to the assay mixtures. MIC values were averages of three independent tests.

When BSA was added to protein-free CDM, a steady decrease in the antimicrobial activity of EGCG was observed as the concentration of BSA increased. The MIC of EGCG against* S. mutans* UA159 cells in the presence of 0.4 mg/mL BSA was 312.5 *μ*g/mL, which was 16 times higher than that observed in the BSA-free CDM control (Figure 2). It suggested that protein present in the media would influence the measurement of MIC.

When the antimicrobial effect of grape seed extract (GSE) was examined, it inhibited growth of most of the test bacteria in CDM (MICs ranging from 62.5 *μ*g/mL to 250 *μ*g/mL), but not when BHI was used (Table 1). The MIC values determined in BHI were up to 16 times greater than those obtained in CDM (Figure 1). GSE did not inhibit growth of test* Candida* species nor did it affect the viability of all test microorganisms (Tables 2–4). Differences in media used did not significantly affect antimicrobial activity assessments of berberine chloride, methyl salicylate, and chlorhexidine gluconate. Comparable MIC and MBC values were noted in both media (Tables 1–4, Figure 1).

## 4. Discussion

The antimicrobial activity of plant-derived polyphenolic compounds has been well-recognized [1–3, 5, 29]. Tea catechins, particularly epigallocatechin gallate (EGCG), as the active antimicrobial compounds in tea infusions, have demonstrated potential in the reduction of dental caries [5, 10, 15, 22, 30–32].

In the screening for or evaluation of the antimicrobial activity of agents, especially plant-derived polyphenolic compounds, complex growth media are routinely used in the microdilution method, due to convenience and their ability to promote rapid growth of test bacteria [17, 33, 34]. Over the years, we have noticed significant variability in efficacy reported by different investigators, potentially due to different choices of growth media used, and found that the antibacterial activity of Oolong tea extract (OTE) against* S. mutans* was reduced in BHI and that pretreatment of OTE with BSA also reduced its antibacterial activity [17, 21, 33, 35]. In our current study, we have also demonstrated differences in the MICs of EGCG against* S. mutans,* depending on the type of growth medium used for the antimicrobial evaluation (Table 1). The MIC and MBC values of EGCG in complex media were significantly higher than those in protein-free chemically defined media. Although less effective than EGCG, GSE, which is rich in the catechin polymer proanthocyanidins, exhibited similar discrepancies in antimicrobial efficacy against most of the test microorganisms when tested in different growth media (Table 1). Unlike flavonoids such as EGCG and proanthocyanidins, we found that the MICs or MBCs of the other nonpolyphenolic test compounds, that is, berberine, methyl salicylate, and chlorhexidine gluconate, were not significantly different when tested in either a complex or chemically defined growth medium. This may have been due to their nonreactivity with the proteins present in the BHI medium. Since polyphenolic compounds including EGCG have been reported to combine with proteins [36–38], we suspected that proteins present in the complex medium such as BHI may have bonded to EGCG, thus reducing its availability to inhibit growth of test microorganisms and contributing to the discrepancies noted in our study and those of previous researchers. Our finding that the addition of BSA to the protein-free CDM decreased the antimicrobial efficacy of EGCG further supported our hypothesis; that is, the discrepancy in antimicrobial activities of plant-derived polyphenolic compounds assessed by in vitro microdilution antimicrobial assays may have been caused by interactions between these test compounds and proteins present in the complex growth medium.

Because the differences in the substrate of growth medium may also affect the susceptibility of microorganisms to the same antimicrobial compound, one may argue that the observed significant fold change in the MICs of polyphenolic compounds might be attributed not to their potential interaction with protein, but rather to the substrate difference of the growth medium. However, this may not be the case, because those nonpolyphenolic compounds tested in this study demonstrated no significant variation in antimicrobial activity in chemically defined or complex media. This could have been due to their inability to bind or precipitate proteins.

## 5. Conclusions

In conclusion, the current study has demonstrated that the presence of proteins in the assay mixtures may significantly affect the outcome when the antimicrobial activity of plant-derived polyphenolic compounds is tested. A nonproteinaceous chemically defined medium should be the assay medium of choice to provide consistent and reliable antimicrobial data and appropriately represent the true test antimicrobial compound. The study also demonstrated the need for standardized methods for the evaluation of the antimicrobial properties of polyphenolic compounds.

## Figures and Tables

**Table 1:** Effects of EGCG and the other test agents on the growth of selected oral bacteria.

Strains	MIC (*µ*g/mL)									
	EGCG		Grape seed extract		Berberine chloride		Methyl salicylate^#^		Chlorhexidine gluconate	
	CDM	BHI	CDM	BHI	CDM	BHI	CDM	BHI	CDM	BHI
*S. mutans*	78.1	625	125	>500	125	125	>3%	>3%	2.5	5
ATCC 25175										
*S. mutans*	39.1	625	125	>500	125	125	>3%	>3%	2.5	5
UA159										
*S. mutans*	78.1	625	62.5	>500	125	250	3%	>3%	5	5
Ingbritt										
*S. gordonii*	≤9.8	625	>500	>500	125	250	0.75%	>3%^*∗*^	10	10
ATCC 49818										
*S. sanguinis*	≤9.8	312.5	>500	>500	125	250	>3%	>3%	10	10
ATCC 10556										
*S. sobrinus*	19.5	1250	62.5	>500	62.5	125	3%	>3%	5	5
ATCC 6715										
*A. actinomycetemcomitans*	39.1	156.3	250	>500	125	250	0.75%	0.75%	5	10
ATCC 43718										
*E. faecalis*	39.1	>1250	62.5	>500	500	>500	>3%	>3%	20	20
ATCC 29212										

^#^The unit of MIC values is % of methyl salicylate (vol/vol).

**Table 2:** Effects of EGCG and the other test agents on the growth of selected *Candida* species in different growth media.

Strains	MIC (*µ*g/mL)									
	EGCG		Grape seed extract		Berberine chloride		Methyl salicylate^#^		Chlorhexidine gluconate	
	RPMI 1640	SDB	RPMI 1640	SDB	RPMI 1640	SDB	RPMI 1640	SDB	RPMI 1640	SDB
*C. albicans *	1250	>1250	>500	>500	>500	500	1.50%	0.75%	6.25	12.5
SC5314										
*C. albicans*	1250	1250	>500	>500	500	500	1.50%	1.50%	3.12	12.5
ATCC 10231										
*C. glabrata *	312.5	>1250	>500	>500	500	500	0.75%	0.38%	3.12	3.12
ATCC 66032										
*C. tropicalis *	≥1250	1250	>500	>500	125	125	3%	0.75%	1.56	0.78
ATCC 13803										
*C. parapsilosis*	1250	>1250	>500	>500	>500	500	0.19%	0.38%	3.12	6.25
ATCC 22019										
*C. kefyr*	156.3	1205	>500	>500	62.5	62.5	0.38%	0.38%	0.78	0.78
ATCC 46764										
*C. krusei*	78.1	>1250	>500	>500	125	125	0.75%	0.75%	1.56	1.56
ATCC 14243										

^#^The unit of MIC values is % of methyl salicylate (vol/vol).

**Table 3:** Effects of EGCG and the other test agents on the viability of selected oral bacteria.

Strains	MBC (*µ*g/mL)									
	EGCG		Grape seed extract		Berberine chloride		Methyl salicylate^#^		Chlorhexidine gluconate	
	CDM	BHI	CDM	BHI	CDM	BHI	CDM	BHI	CDM	BHI
*S. mutans*	156.3	1250	500	>500	125	125	>3%	>3%	5	10
ATCC 25175										
*S. mutans *	78.1	1250	>500	>500	125	125	>3%	>3%	5	10
UA159										
*S. mutans*	78.1	1250	500	>500	250	250	3%	>3%	5	20
Ingbritt										
*S. gordonii *	≤9.77	1250	>500	>500	250	250	0.75%	>3%	10	10
ATCC 49818										
*S. sanguinis *	19.5	>1250	>500	>500	125	250	>3%	>3%	10	10
ATCC 10556										
*S. sobrinus *	78.1	>1250	125	>500	62.5	125	>3%	>3%	5	5
ATCC 6715										
*A. actinomycetemcomitans*	39.1	156.3	>500	>500	250	500	0.75%	0.75%	5	10
ATCC 43718										
*E. faecalis*	625	>1250	>500	>500	>500	>500	>3%	>3%	20	20
ATCC 29212										

^#^The unit of MBC/MFC values is % of methyl salicylate (vol/vol).

**Table 4:** Effects of EGCG and the other test agents on the viability of selected *Candida* species in different growth media.

Strains	MFC (*µ*g/mL)									
	EGCG		Grape seed extract		Berberine chloride		Methyl salicylate^#^		Chlorhexidine gluconate	
	RPMI 1640	SDB	RPMI 1640	SDB	RPMI 1640	SDB	RPMI 1640	SDB	RPMI 1640	SDB
*C. albicans *	>1250	>1250	>500	>500	>500	>500	1.50%	0.75%	6.25	12.5
SC5314										
*C. albicans*	>1250	>1250	>500	>500	>500	>500	1.50%	1.50%	6.25	12.5
ATCC 10231										
*C. glabrata *	>1250	>1250	>500	>500	>500	>500	0.75%	0.38%	3.12	6.25
ATCC 66032										
*C. tropicalis *	>1250	1250	>500	>500	125	125	3%	1.50%	1.56	1.56
ATCC 13803										
*C. parapsilosis *	>1250	>1250	>500	>500	>500	>500	0.19%	0.38%	3.12	12.5
ATCC 22019										
*C. kefyr *	>1250	1250	>500	>500	62.5	62.5	0.38%	0.38%	0.78	1.56
ATCC 46764										
*C. krusei *	>1250	>1250	>500	>500	125	125	0.75%	0.75%	1.56	1.56
ATCC 14243										

^#^The unit of MBC/MFC values is % of methyl salicylate (vol/vol).
